# Oxidative stress activates the transplanted adipose-derived stem cells to exert antioxidant effects in alopecia treatment

**DOI:** 10.1080/13510002.2025.2503128

**Published:** 2025-06-06

**Authors:** Xuer Sun, Minliang Chen

**Affiliations:** Senior Department of Burns and Plastic Surgery, The Fourth Medical Center of PLA General Hospital, Beijing, People’s Republic of China

**Keywords:** Oxidative stress, mesenchymal stem cell, nanofat, alopecia, emulsified fat, fat transplantation, hair follicle, Nrf2

## Abstract

**Background:**

Alopecia is a global dermatological challenge. Adipose-derived stem cells (ADSC) show therapeutic potential, but their mechanisms in promoting hair regrowth, particularly under oxidative stress conditions, remain unclear..

**Objective:**

To investigate ADSC's role in promoting hair regrowth by mitigating oxidative stress.

**Methods:**

Using H₂O₂-stressed HaCaT cells, ADSC's protective effects were evaluated via conditioned medium (CM) and co-culture. Assessments included cell viability, colony formation, ROS, MDA, antioxidant enzymes, and 8-OHdG. Nrf2 activation was analyzed by immunofluorescence and Western blot. A mouse radiation injury model validated findings.

**Results:**

Non-pretreated ADSC offered limited oxidative protection to HaCaT cells. Conversely, H₂O₂-pretreated ADSC significantly enhanced HaCaT viability and proliferation in both CM and co-culture systems. This involved paracrine activation of the Nrf2 pathway in HaCaT cells, boosting antioxidant enzymes, accelerating ROS clearance, and reducing lipid peroxidation. These effects were reversible with Nrf2 inhibition. In vivo, CM from H₂O₂-stimulated ADSC promoted hair regrowth in irradiated mice, outperforming CM from non-pretreated ADSC by activating Nrf2 and reducing tissue oxidative damage.

**Conclusions:**

Oxidative stress potentiates the protective capacity of ADSC against oxidative via Nrf2-dependent paracrine mechanisms, offering a promising strategy for alopecia treatment.

## 1. Introduction

Alopecia is a prevalent condition that affects individuals across different regions, ethnicities, and genders. The incidence of the most common type, androgenetic alopecia (AGA), exceeds 50% in men over the age of 50, making it a significant global health concern [1]. Alopecia not only impacts physical appearance but also compromises patients’ psychological well-being and social functioning, contributing to elevated stress levels and psychological comorbidities [2]. All the current treatments, including drug therapy (such as oral finasteride and topical minoxidil), hormone therapy, immunotherapy, phototherapy, and hair transplantation, have limitations, such as suboptimal efficacy, high relapse rates after treatment cessation, adverse drug reactions, toxicity, and procedural trauma [3]. As a result, the treatment of alopecia remains challenging and unresolved globally.

In recent years, nanofat grafting has attracted considerable attention in plastic surgery [4–6]. Over the past decade, it has also been investigated for its potential in promoting hair regrowth. Although this application is nascent, accumulating evidence from cellular studies [7,8], preclinical models [9,10], clinical case series [11–16], randomized double-blind controlled trials [17], and systematic reviews [18] support its therapeutic potential. Therefore, nanofat and adipose-derived stem cells (ADSC) hold significant potential in the treatment of alopecia.

Despite these promising results, the underlying mechanisms of their effectiveness remain unclear. The prevailing theory is that mesenchymal stem cells (MSC) secrete paracrine growth factors, which promote the proliferation and regulate the cell cycle of hair matrix keratinocytes (HMKs) and dermal papilla cells (DPC) [19]. However, this explanation does not fully address the pathogenesis of alopecia.

Different types of alopecia have distinct etiologies and pathogeneses, but oxidative stress is a common pathological basis in nearly all forms of hair loss [20,21]. In hair follicles (HFs), reactive oxygen species (ROS) can be generated through various physiological and pathological processes, including cellular metabolism, malnutrition, trauma, inflammation, hormonal activity, environmental factors, genetics, microbiota, and aging [22–25]. The body has endogenous defense mechanisms against ROS, but when these systems fail to maintain redox balance, excessive ROS accumulation leads to cellular damage.

Research has demonstrated that ROS impairs the proliferation and migration of HF cells, induces negative regulators of hair growth such as TGF-β, prolongs the resting phase, shortens the growth phase, and accelerates the premature aging of HF cells [25, 26]. Additionally, ROS oxidizes lipids, proteins, and DNA, causing destruction to cell membranes and organelles, ultimately leading to programmed cell death [27]. The relationship between ROS and the development of AGA [28–32]and alopecia areata (AA) [33–36]has been well established. Furthermore, controlling oxidative stress has been shown to significantly improve hair health [37,38].

While various studies suggest that ADSC can activate the Nrf2 signaling pathway to protect tissues from oxidative stress, there is currently no direct evidence explaining how ADSC promote scalp hair regrowth under pathological conditions through their antioxidative properties.

## 2. Materials and methods

### 2.1. Nanofat harvesting and grafting

All experimental procedures were approved by the Clinical Trial Ethics Committee of the Fourth Medical Centre, Chinese People’s Liberation Army General Hospital (Registration Number: 2024YL039-KS001). Patients diagnosed with AGA provided informed consent. Nanofat was harvested via autologous abdominal liposuction, following the established method [39]. Briefly, lipoaspirate was washed and mechanically emulsified by passing the fat through a 0.8-mm nanometer transverter. After 30 passes, the fat became emulsified. The fatty liquid was then filtered through sterile nylon cloth to remove connective tissue remnants, and the filtrate was collected in a sterile container. The injection volume was 1.0 mL/cm² of scalp, administered to the alopecia-affected areas.

### 2.2. Scalp sampling and preparation

Scalp tissues from healthy individuals with head trauma were used as the negative control group, while the control group consisted of alopecia patients. Scalp biopsy tissues were collected from alopecia patients treated with ADSC. Samples were washed, fixed in 4% paraformaldehyde for 24 hours, dehydrated, and embedded in paraffin. Sections were cut at a thickness of 5 µm using a rotary microtome (Leica, Wetzlar, Germany).

### 2.3. Cell culture and grouping

The HaCaT cell line was generously provided by Professor Jin from the Department of Experimental Hematology and Biochemistry, Beijing Institute of Radiation Medicine, Beijing, China. ADSC isolation and culture were performed using the standard method [40]. The lipoaspirate was washed, cut into pieces, and digested with collagenase (BioFroxx, Guangzhou, China), followed by centrifugation to discard the supernatant. The resulting pellet was resuspended and cultured in DMEM/F12 medium (Meilunbio, Dalian, China) supplemented with 10% fetal bovine serum (Excell, Shanghai, China). Medium was changed every other day, and cells were passaged when they reached 80% confluence.

Conditioned medium (ADSC-CM) was obtained from passage 5–6 ADSC. For H_2_O_2_ pretreatment, 0.1 mM H_2_O_2_ was added for 24 hours, followed by a medium change. After another 24 hours, the medium was collected as pretreated ADSC conditioned medium (pCM).

Cells were seeded in 6-well plates at an density of 3 × 10^5^ cells/well. Once the cells adhered to the well surfaces, different groups of media were added as shown in Table 1. Samples were collected 24 hours after changing the media.

**Table 1.** Cell grouping and medium component.

Groups	Medium component
Control (Con)	DMEM/F12 (Meilunbio, Dalian, China) + 5% serum (Excell, Shanghai, China)
Pretreated Conditioned medium (pCM)	50% DMEM/F12 + 50% pCM + 5% serum
Brusatol (Bru)	Con + 40 nM Bru (MedChemExpress, New Jersey, US)
H_2_O_2_ (H)	Con + 0.4 mM H_2_O_2_
H_2_O_2_ + Pretreated Conditioned medium (H + pCM)	pCM + 0.4 mM H_2_O_2_
H_2_O_2_ + Pretreated Conditioned medium + Brusatol (H + pCM + B)	H + pCM + 40 nM Bru

### 2.4. Co-culture and cloning formation

Transwell co-culture plates (Corning, New York, USA) were used. ADSC (250,000 cells) were seeded in the upper compartment, while HaCaT cells (4000 cells) were seeded in the lower compartment. On the second day, 0.1 mM of H_2_O_2_ was added to the culture medium to induce oxidative stress. The medium was changed every other day, and photos were taken on the 7th day.

### 2.5. Animal experiments

Ten-week-old Balb/c mice were purchased from HFK Bioscience Inc. (Beijing, China) and housed in air-conditioned rooms at a constant temperature of 21 ± 2°C with a 12-hour light/dark cycle. Mice were provided with regular lab chow and water ad libitum throughout the experiments. All experimental procedures were approved by the Institutional Animal Care and Use Committee of Chinese People’s Liberation Army General Hospital, Beijing, China (Registration Number: 2022-X18-03).

The mice were shaved on their backs and randomly divided into four groups (n = 3):

1. Control group (Con): normal mice
2. Irradiation group (IR): 16 Gy X-ray irradiation
3. CM treatment group (IR + CM): 16 Gy X-ray irradiation + CM injection
4. pCM treatment group (IR + pCM): 16 Gy X-ray irradiation + pCM injection

Subcutaneous injections (100 µl/site) were administered at four dorsal sites every 5 days.

### 2.6. CCK-8 assay

Cell viability was measured using the Cell Counting Kit-8 (Yeasen, Shanghai, China) according to the manufacturer’s instructions. A total of 2000 cells per well were seeded in a 96-well plate. Once the cells adhered, different groups of media were added. After 24 hours, 10 μL of CCK-8 solution was added to each well, followed by a 2-hour incubation at 37°C in the dark. Absorbance was measured at 450 nm.

### 2.7. Antioxidant enzyme activity assay

Levels of total glutathione (GSH), malondialdehyde (MDA), and superoxide dismutase (SOD) were measured using commercial detection kits (Beyotime, Shanghai, China) following the manufacturer’s protocols. Absorbance was measured at 412, 532, and 450 nm, respectively, and calculated according to the provided formulas.

### 2.8. Immunofluorescence Staining

Tissue sections were deparaffinized and boiled in citrate antigen retrieval buffer for 10 minutes. Cell samples were fixed with 4% paraformaldehyde for 30 minutes and permeabilized with 0.3% Triton X-100 for 5 minutes. Both types of samples were blocked with 10% normal horse serum, followed by overnight incubation with primary antibodies (rabbit anti-8OHdG, Bioss, Beijing, China; rabbit anti-Nrf2, Proteintech, Wuhan, China). Secondary antibody incubation and DAPI staining for nuclear localization were then performed. Images were captured using a fluorescence microscope (Soptop, Yuyao, China), and staining quantification was done using ImageJ software (National Institutes of Health, Bethesda, MD, USA).

### 2.9. ROS measurement

Reactive oxygen species (ROS) levels were measured using a commercial assay kit (Meilunbio, Dalian, China). DCFH-DA (2,7-dichlorodihydrofluorescein diacetate) was diluted to a final concentration of 10 μM and added to the cells, followed by a 30-minute incubation at 37°C in the dark. After the DCFH-DA solution was discarded, different groups of media were added. ROS fluorescence was detected at 488 nm after 60 minutes of incubation.

### 2.10. Western blot

Protein lysates were prepared using RIPA buffer (CwBio, Taizhou, China) with protease and phosphorylase inhibitors (CwBio, Taizhou, China). The lysates were repeatedly aspirated to ensure complete cell lysis. After 5× loading buffer was added, the samples were heated at 100°C for 10 minutes to denature the proteins that were separated by SDS-PAGE and transferred onto NC membranes, which were blocked with 5% non-fat milk for 1 hour before overnight incubation at 4°C with primary antibodies (rabbit anti-Nrf2, Proteintech, Wuhan, China; NQO1, Abcam, Cambridge, UK; Actin, Santa Cruz, Texas, USA). After being washed with TBST, the membranes were incubated with secondary antibodies for 1 hour at room temperature, followed by further TBST washes. Protein bands were visualized using enhanced chemiluminescence solution (Thermo Fisher Scientific, Waltham, USA) and captured using a Tanon Imaging System (Tanon, Shanghai, China).

### 2.11. Statistical analysis

Statistical analyses were performed using SPSS software. Data were presented as mean ± standard error of the mean (SEM). Normality tests for proliferative viability, MDA, SOD, GSH, GSSG, GSH/GSSG ratio across groups were performed using the Shapiro–Wilk method combined with Q-Q plots. Homogeneity of variance was assessed using Levene test. The results indicated that the aforementioned data conformed to both normal distribution and homogeneity of variance. Consequently, one-way ANOVA was selected to compare intergroup differences, followed by pairwise comparisons and adjustment for multiple comparisons using Tukey’s HSD method. Normality tests for 8-OHdG, TUNEL, Nrf2 immunofluorescence quantification data, and relative protein quantification data of Nrf2, NQO1, and HO-1 across groups were performed using the Shapiro–Wilk method combined with Q-Q plots. Homogeneity of variance was assessed using Levene test. The results indicated that the aforementioned data followed a normal distribution but did not meet the assumption of homogeneity of variance. Consequently, Welch ANOVA was employed to compare intergroup differences, followed by pairwise comparisons and adjustment for multiple comparisons using the Games-Howell test. The following notations were used to indicate significance levels: ns (*P* > 0.05), * (*P* < 0.05), ** (*P* < 0.01), *** (*P* < 0.001), and **** (*P* < 0.0001).

## 3. Results

### 3.1. Oxidative stress in patients’ scalp and effectiveness of nanofat treatment in clinical cases

**Figure 1.**
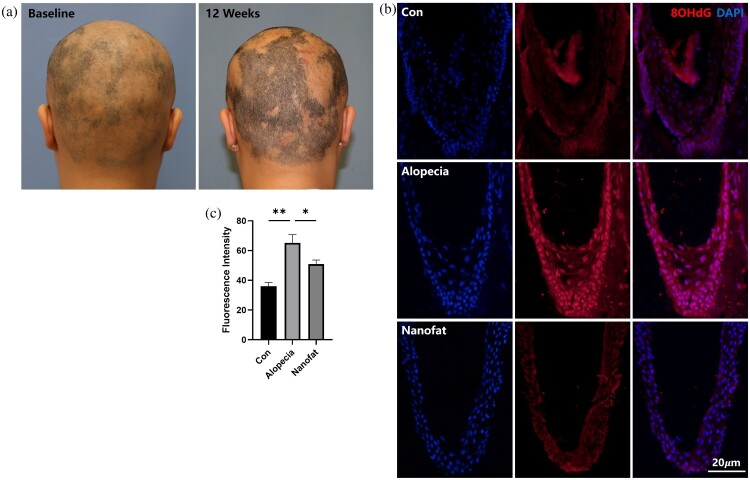
Effectiveness of nanofat treatment in clinical alopecia cases. (a) Photographs taken before and after nanofat treatment in a patient with androgenetic alopecia. (b, c) Immunofluorescent staining of 8OHdG (a marker of DNA oxidative stress damage) in scalp tissues from healthy individuals (Con) and alopecia patients, with or without nanofat treatment (Nanofat/Alopecia).

After two cycles of autologous ADSC injection (July 2021 and January 2022), significant improvements in hair density and thickness were observed in the top, occipital, and temporal regions compared to the baseline (Figure 1(A)), with high levels of patient satisfaction reported.

In alopecia patients, oxidative stress damage was evident in hair follicles (HF), as indicated by an increased number of 8OHdG-positive cells in the scalp tissue compared to healthy individuals. After nanofat treatment, the number of 8OHdG-positive cells markedly decreased (Figure 1(B,C)).

### 3.2. Co-culture with ADSC alleviated oxidative stress damage in HaCaT cells caused by H_2_O_2_

Since keratinocytes are the primary cell type in hair follicles, HaCaT cells were used as an *in vitro* model. H_2_O_2_ was used to induce oxidative stress damage in HaCaT cells, and the protective effects were established by co-culturing HaCaT cells with ADSC.

**Figure 2.**
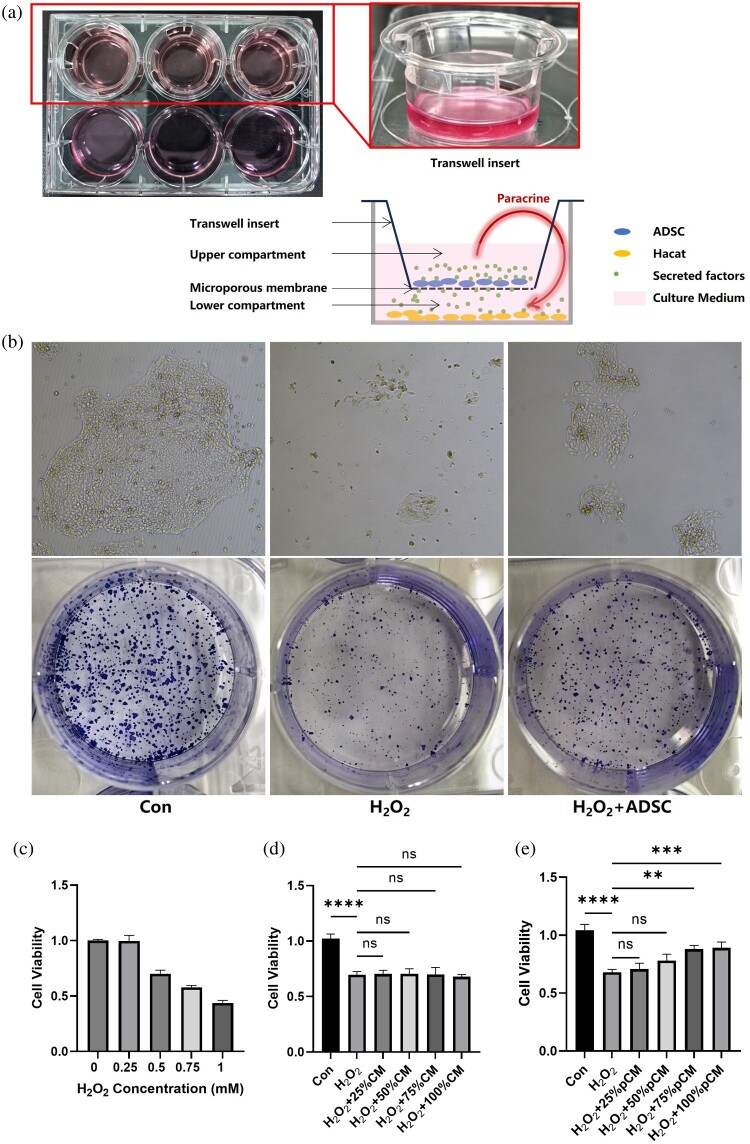
ADSC's effect on reducing H_2_O_2_-induced oxidative stress in HaCaT cells. (a) Photograph and schematic representation of the Transwell co-culture system. (b) Microscopic and macroscopic images of the colony formation assay. (c) Changes in HaCaT cells viability at different H_2_O_2_ concentrations. (d) Antioxidative effects of non-pretreated ADSC-conditioned medium (ADSC-CM) on H_2_O_2_-treated HaCaT cells. (e) Antioxidative effects of pretreated ADSC-CM on HaCaT cells. CM: ADSC-conditioned medium; pCM: pretreated ADSC-conditioned medium.

In the co-culture system, a Transwell insert with a microporous membrane was suspended in the well, ensuring that factors secreted by cells in the upper compartment could pass freely to cells in the lower compartment. ADSC were seeded in the upper compartment while HaCaT cells were seeded in the lower one. H_2_O_2_ (0.1 mM) was added to induce oxidative stress. A colony formation assay was used to assess the antioxidative effects of ADSC (Figure 2(A)).

After H_2_O_2_ addition, cells appeared damaged and deformed, with cell proliferation nearly halted. Colony formation was significantly inhibited by H_2_O_2_, and large colonies with more than 20 cells were rarely observed by day 7. However, ADSC co-culture restored colony formation capacity, yielding colonies comparable in size and number to the untreated control group (Figure 2(B)).

### 3.3. Normal ADSC had limited effect in reducing H_2_O_2_-induced cytotoxicity in HaCaT cells

To further quantitatively assess the antioxidant stress effects of ADSC, we employed conditioned medium of ADSC to protect HaCaT cells against oxidative stress damage and subsequently measured the viability of the HaCaT cells.

We began by assessing the cytotoxic effects of various H_2_O_2_ concentrations on HaCaT cells. Cells were treated with 0.25, 0.5, 0.75, and 1 mM H_2_O_2_ for 24 hours. Except for the 0.25 mM concentration, which did not alter cell viability, the other three concentrations reduced cell viability to 70%, 50%, and 30%, respectively (Figure 2(C)). To balance detectable cell damage with potential recovery, we selected 0.5 mM H_2_O_2_ as the optimal concentration for subsequent experiments, as it reduced cell activity to about 70% of the normal state.

Regardless of the concentration used (25%, 50%, 75%, 100%), conditioned medium from normal ADSC showed minimal protective effect against H_2_O_2_-induced oxidative stress in HaCaT cells (Figure 2(D)).

### 3.4. H_2_O_2_ pretreatment actatived ADSC’ protective effects in HaCaT cells against H_2_O_2_-induced cytotoxicity

The possible reasons are considered as follows: First, the conditioned medium lacks ADSC cells, resulting in a limited and insufficient concentration of secreted factors to sustain their protective effects. Second, the concentration of secreted factors from ADSC is adequate, but compared to the co-culture system, since ADSC are not exposed to H_2_O_2_, their protective capacity is not ‘activated.’

To test this, we pretreated ADSC with 0.1 mM H_2_O_2_ for 24 hours, followed by medium change, and after another 24 h later, the conditioned medium was collected for subsequent experiments, named pretreated ADSC conditioned medium (pCM). pCM was used as the conditioned medium for subsequent experiments.

We found that in HaCaT cells, treatment with 0.5 mM H₂O₂ for 24 hours reduced cell viability to 70%. However, after adding the conditioned medium from pretreated ADSC (pCM), the cell viability increased to approximately 90% (Figure 2(E)).

Therefore, we conclude that under normal conditions, the paracrine factors of ADSC do not possess antioxidant stress capabilities. However, upon stimulation with H₂O₂, ADSC acquire the ability to counteract oxidative stress and protect the viability and proliferative capacity of HaCaT cells through paracrine mechanisms.

### 3.5. pCM ameliorated the oxidative stress state of HaCaT cells under hydrogen peroxide stimulation

**Figure 3.**
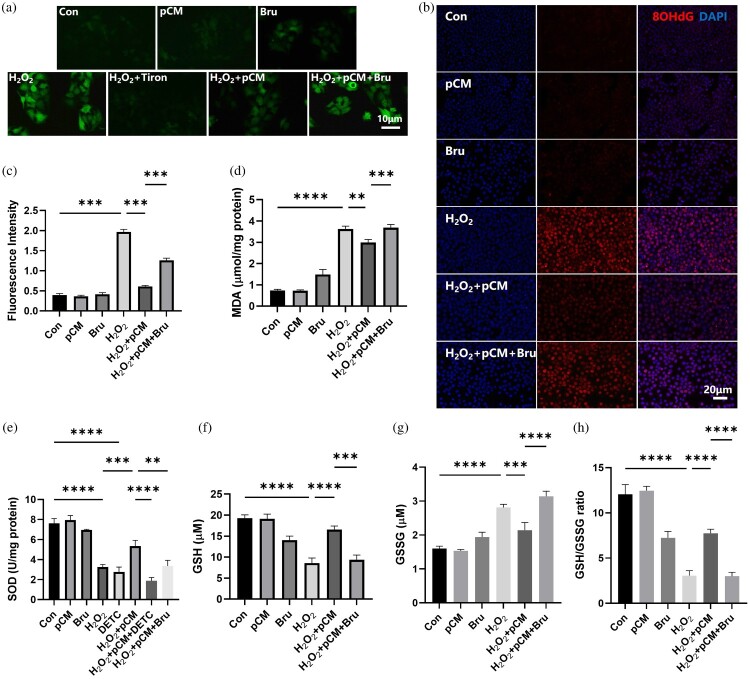
Oxidative changes observed in different experimental groups. (a) ROS production. (b-c) 8OHdG expression, a marker of DNA oxidative stress damage. (d) Malondialdehyde (MDA) content, an index of lipid peroxidation. (e) Superoxide dismutase (SOD) activity. (f) Glutathione (GSH) content. (g) Glutathione disulfide (GSSG) content. (h) GSH/GSSG ratio. pCM: pretreated ADSC-conditioned medium; Bru: Brusatol, a specific Nrf2 signaling pathway inhibitor.

Then, the oxidative stress status of the cells was assessed. Under normal conditions, the intracellular levels of reactive oxygen species (ROS) were low. However, after treatment with 0.4 mM H₂O₂ for 24 hours, ROS levels were significantly upregulated. 1 mM Trion (as a ROS inhibitor) was added to another H₂O₂-treated group, resulting in an immediate decrease in ROS levels to baseline values. This observation confirmed the specificity of the probe (Figure 3(A)). ROS accumulation induces cellular damage through multiple mechanisms. Malondialdehyde (MDA) and 8-hydroxy-2'-deoxyguanosine (8-OHdG) were critical markers for lipid peroxidation and DNA oxidative stress damage, respectively. Under normal conditions, the expression levels of MDA and 8-OHdG are relatively low. However, after 24 hours of treatment with H₂O₂, MDA expression increased to 4.9 times its original level, and 8-OHdG showed positivity in a large number of cells (Figure 3(B–D)). When oxidative stress damage occurs, the human body initiates a series of antioxidant defenses to counteract ROS-induced damage. However, excessive ROS can inhibit the activity of antioxidant enzymes. Following H₂O₂ treatment, the activity of superoxide dismutase (SOD) decreased to 42%. Reduced glutathione (GSH) was also depleted to 45% of baseline levels, with a significant portion oxidized into glutathione disulfide (GSSG), resulting in a decline of the GSH/GSSG ratio to 26% of normal values. After pCM intervention, the antioxidant system was reactivated: SOD activity recovered to 70%, though this increase could be blocked by the SOD inhibitor DETC (100 μM). Concurrently, GSSG was reduced back to GSH, restoring GSH content to 85% of baseline and elevating the GSH/GSSG ratio to 64% of normal levels (Fig 3E-H). Alongside these changes, ROS levels significantly decreased, and the extent of lipid peroxidation and DNA oxidative stress damage was reduced by 17.7% and 69%, respectively (Fig 3A-D).

These results indicate that the endogenous antioxidant stress system activity in HaCaT cells was suppressed after H₂O₂-induced damage. However, the conditioned medium from H₂O₂-pretreated ADSC was able to restore the activity of the antioxidant system, thereby reducing cellular oxidative stress damage.

### 3.6. pCM activated the Nrf2 signaling pathway, and Nrf2 inhibition impaired its protective effects

**Figure 4.**
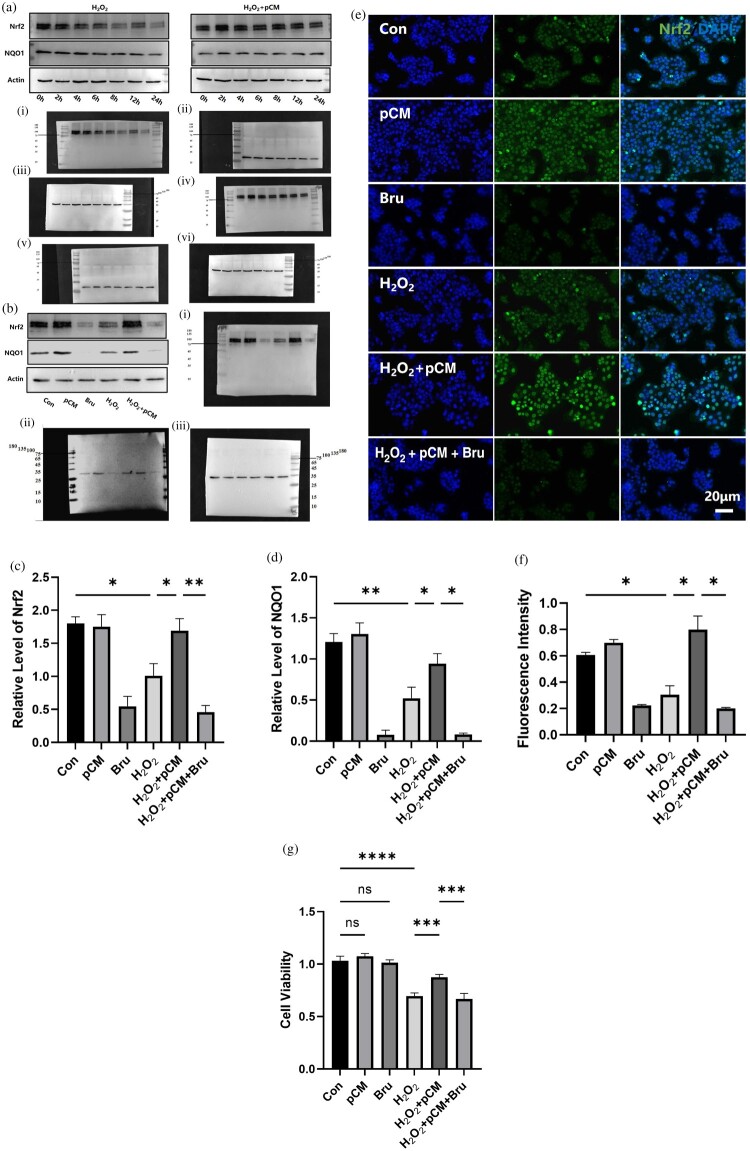
Detection of Nrf2 signaling pathway activation. (a) Time course of Nrf2 pathway activation by H_2_O_2_ and pCM. (b–d) Comparison of Nrf2 activation levels across different groups. (e, f) Immunofluorescent staining of Nrf2 protein in various groups. (g) Cell viability in the different experimental groups. pCM: pretreated ADSC-conditioned medium; Bru: Brusatol, a specific Nrf2 signaling pathway inhibitor.

The Nrf2 signaling pathway is a key mechanism for antioxidative stress, activating various endogenous antioxidant defenses. We first examined the activation pattern of the Nrf2 pathway in HaCaT cells following H_2_O_2_ treatment. Samples were collected at 0, 2, 4, 6, 8, 12, and 24 hours post-H_2_O_2_ exposure. Under unstimulated conditions, Nrf2 protein was partially expressed. However, within 2 hours of H₂O₂ treatment, the expression of Nrf2 protein began to decline, with a significant reduction by 4 hours, reaching a lower level by 8 hours, and remaining at this low level at 24 hours. The downstream NQO1 protein levels also decreased, albeit with a slight delay and to a lesser extent compared to Nrf2. However, when the preconditioned ADSC conditioned medium was added simultaneously with H₂O₂, the levels of Nrf2 protein remained stable and even slightly increased, while the expression of the downstream NQO1 protein also showed a moderate increase (Figure 4(A)).

We further examined HaCaT cells in different groups using immunofluorescence and Western blot methods. The results showed that under normal conditions, Nrf2 protein was partially expressed. After the addition of H₂O₂, the expression of Nrf2 decreased, and it translocated from the nucleus to the cytoplasm. However, upon the addition of pCM, the expression of Nrf2 protein increased, and its nuclear translocation became more pronounced (Figure 4(B–F)).

To further demonstrate that the ADSC conditioned medium exerts its oxidative protective effects by activating the Nrf2 signaling pathway, we used a specific Nrf2 pathway inhibitor, Brusatol, to block this pathway and observed whether the protective effects were abolished. The results showed that after treatment with Brusatol alone, the expression of Nrf2 protein significantly decreased. Moreover, Brusatol effectively blocked the upregulation of Nrf2 protein induced by pCM (Figure 4(B–F)). When the upregulation of Nrf2 protein by pCM was inhibited, the protective effects of pCM on cell proliferation and viability were nearly eliminated (Figures 3(A–G) and Figure 4(G)).

### 3.7. ADSC promoted hair regeneration in irradiated mouse skin

**Figure 5.**
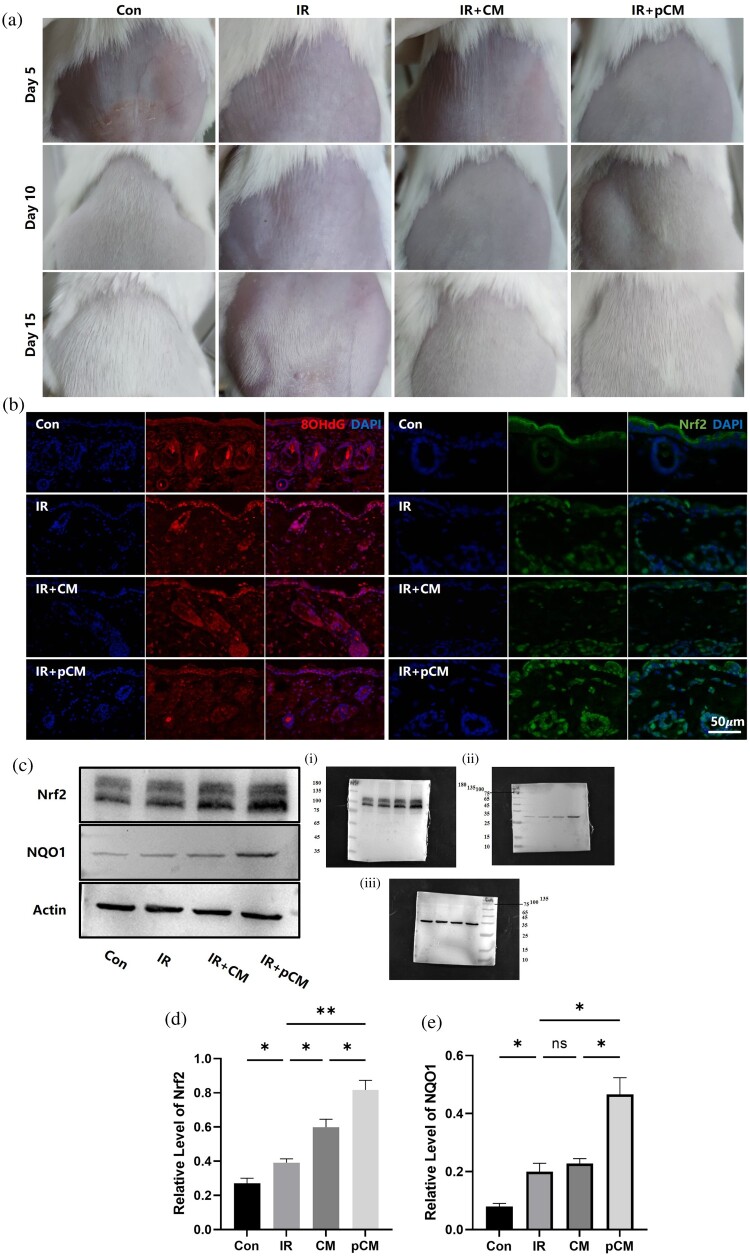
Antioxidative effects of ADSC on irradiated mouse skin. (a) Macroscopic images of hair regeneration in different groups of mice. (b) Immunofluorescent staining showing 8OHdG and Nrf2 expression and localization in mouse skin. (c–e) Nrf2 and NQO1 expression in mouse skin across different groups. IR: irradiated; pCM: pretreated ADSC-conditioned medium.

10-week-old Balb/c mice had their back hair removed and were irradiated with X-rays to induce oxidative stress in the skin. Hair regeneration was then observed. In the control group, uniform short hair was visible by day 10, turning the skin white. By day 15, hair grew several millimeters. In contrast, X-ray irradiation delayed or inhibited hair regeneration. On day 10, no hair growth was observed, and by day 15, hair growth was uneven, with only partial whitening of the skin. Subcutaneous injection of pCM accelerated hair regeneration, with uniform hair growth observed by day 15 as in the control group. Mice injected with non-pretreated ADSC-CM showed only partial improvement in hair regeneration (Figure 5(A)).

The dorsal skin of mice was subjected to assess oxidative stress damage and the expression and nuclear translocation of Nrf2 protein. Under normal conditions, 8OHdG was almost negative in the nuclei of various cell types in the skin. After X-ray irradiation, the nuclei of hair follicles and epidermal cells showed significant positivity for 8OHdG, with a slight increase in Nrf2 protein expression and partial nuclear translocation. Following subcutaneous injection of pCM, the number of 8OHdG-positive cells significantly decreased, and the intensity of the positive signal was markedly reduced. Concurrently, Nrf2 protein expression was significantly increased, with a substantial amount of the protein translocating into the nucleus. In contrast, CM that was not pretreated with H₂O₂ did not exhibit these effects (Figure 5(B)). Western blot analysis confirmed that pCM injection upregulated Nrf2 and its downstream antioxidant enzyme NQO1, with less pronounced effects in the ADSC-CM group (Figure 5(C–E)).

## 4. Discussion

This study was conducted following a classic research approach: First, a damage-protection model was established through cell experiments, and the antioxidant stress damage effects of ADSC were observed, along with the requirement of an oxidative stress environment for activation. Next, the oxidative stress status of the cells was evaluated by assessing antioxidant enzyme activity and oxidative stress markers. Furthermore, the activation state of the Nrf2 pathway was examined using experimental methods such as immunofluorescence staining and Western blot, and its involvement was validated using the Nrf2-specific inhibitor brusatol. Finally, animal experiments were performed to further validate the findings. Through this step-by-step and multi-dimensional approach, it was confirmed that the antioxidant stress damage effects of ADSC require activation by an oxidative stress environment, and that Nrf2 plays a role in the antioxidant stress function of ADSC.

In the animal experiment section, considering our focus on constructing a model of hair loss induced by oxidative stress damage, we selected X-ray irradiation to establish a radiation injury model, which resulted in a highly noticeable hair loss effect. For the treatment phase, since antioxidant factors are primarily secreted into the conditioned medium through paracrine mechanisms, we administered the intervention via multi-point injections of the conditioned medium. Additionally, we compared the effects of conditioned medium from ADSC with and without H₂O₂ activation, demonstrating the importance of the H₂O₂ activation step in enabling ADSC to acquire antioxidant stress functionality.

**Figure 6.**
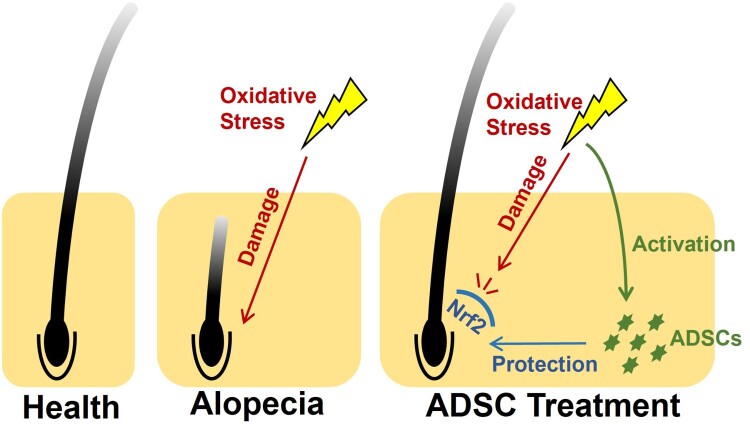
Schematic representation of the study's main concept. Oxidative stress is a critical pathophysiological factor in alopecia development (middle). ADSC promote hair regrowth through antioxidant effects, a process that requires activation by oxidative stress (right).

Current research on the mechanisms of ADSC treatment for alopecia focuses on growth factors and cell cycle regulation [19]. Previous studies have shown that mesenchymal stem cells (MSC) can secrete growth factors such as IGFBP, PDGF, bFGF, EGF, HGF, and VEGF, which activate signaling pathways including Erk, Akt, and Wnt. These pathways promote the proliferation of hair follicle (HF) keratinocytes (HMKs) and dermal papilla cells (DPC), shorten G1 phase cell cycle arrest, extend DNA synthesis and mitosis, and induce HFs into the growth phase. Our study unveils a novel mechanism whereby oxidative stress primes ADSC to promote hair regrowth via Nrf2-mediated antioxidant responses (Figure 6). In other words, it was the oxidative stress in the patient’s scalp that activated transplanted ADSC, enabling them to exert antioxidant functions in the treatment of alopecia.

We found that ADSC could enhance the viability of oxidatively damaged HaCaT cells after pre-treatment with H_2_O_2_, both in a co-culture system and in conditioned medium. Without this activation step, ADSC had minimal impact on promoting the proliferation of damaged cells.

When H_2_O_2_ was added to the conditioned medium, two potential activation factors were considered: H_2_O_2_ itself or factors released by damaged HaCaT cells. Our experiments confirmed that it was the latter. In the CCK8 experiment, we stimulated ADSC with H_2_O_2_ and added the supernatant to the HaCaT cells culture system, without spatial contact between the two cell types.

What happens in ADSC after H_2_O_2_ exposure? H_2_O_2_ induces oxidative stress damage to MSC, disturbing intracellular redox balance and shifting the cells into a state of oxidative stress. This process can lead to premature senescence and telomere shortening [40]. However, MSC can adapt to changes in their surrounding environment. Li et al. found that preconditioning with low concentrations of H_2_O_2_ for a short period protected human umbilical cord MSC (UB-MSC) from long-term exposure to high concentrations of H_2_O_2_ [41]. Wang et al. showed that, compared to a high-concentration H_2_O_2_ injury group, MSC pretreated with low concentrations of H_2_O_2_ had reduced ROS and MDA levels, increased SOD and CAT activities, and reduced mitochondrial damage. Cell viability and survival rates increased, while apoptosis rates decreased [42]. Waheed et al. compared the effects of pulsed and sustained oxidative stress on human ADSC, finding that pulsed exposure induced higher ROS production, cytotoxicity, and inflammatory factor release, while sustained exposure promoted the production of antioxidant enzymes, allowing cells to better adapt to oxidative stress [43].

In addition to adapting to oxidative stress, MSC may acquire new functions. H_2_O_2_ may induce a defensive response in ADSC, protecting both themselves and neighboring HaCaT cells. Our experiments showed that after adding ADSC-conditioned medium, the Nrf2 signaling pathway was activated, conferring protective effects. When a Nrf2 pathway blocker was used, the protective effect disappeared, confirming the pathway’s critical role in this process. Recent studies have demonstrated that MSC, their culture supernatants, and exosomes can exert antioxidant effects by activating the Nrf2 signaling pathway in the treatment of various diseases across multiple systems and organs [44–60]. This highlights the importance of oxidative stress mitigation in ADSC-based therapies.

Several studies have explored the therapeutic potential of H_2_O_2_-pretreated MSC for a variety of conditions. Rahimi et al. found that transplantation of bone marrow-derived MSC (BM-MSC) for spinal cord injury in rats improved locomotor scale scores and motor function recovery, with H_2_O_2_-pretreated MSC increasing local expression of brain-derived neurotrophic factor (BDNF) [61]. Bai et al. showed that human ADSC exosomes significantly promoted the proliferation of human umbilical vein endothelial cells (HUVECs), enhanced cell migration and tube formation, and improved flap survival rates in a rat model of ischemia-reperfusion injury, with greater effects observed after H_2_O_2_ pretreatment [62]. Zhang et al. used human umbilical cord MSC to treat myocardial infarction (MI) in mice, finding that cardiac neovascularization and left ventricular contractility improved, while myocardial fibrosis decreased. These effects were more pronounced in the H_2_O_2_-pretreated group, where MSC increased endothelial cell migration and proliferation, and IL-6 secretion by 25-fold [63]. Boopathy et al. found that after H_2_O_2_ treatment, rat BM-MSC increased mRNA expressions of endothelial-related genes (Flt1, vWF, PECAM1) and early cardiac markers (nkx2-5, αMHC), while decreasing the expressions of early smooth muscle markers and fibroblast markers. This effect was regulated by the Notch1 signaling pathway, which could be disrupted by Notch inhibition [64]. Despite this growing body of evidence, the antioxidative stress function of pretreated ADSC in alopecia has never been reported so far.

In this study, we did not assess the purity of ADSC, as we used nanofat rather than pure ADSC for clinical treatment. Although ADSC constitute a significant portion of nanofat, other cell types are also present and may contribute to its therapeutic effects. By using adherent cells from nanofat, we aimed to more accurately reflect the clinical impact of nanofat treatments.

There are still unresolved problems. First, we do not yet fully understand the mechanisms behind H_2_O_2_-induced activation in ADSC or the specific components they secrete that initiate the Nrf2 signaling pathway. Second, while we focused on oxidative stress, it remains unclear whether ADSC also play a therapeutic role in addressing other factors, such as inflammatory responses, microbial flora, and androgen toxicity – and which of these is most crucial. Third, additional data are needed on changes in ADSC secretion profiles before and after H_2_O_2_ treatment, as well as transcriptional changes in HaCaT cells following exposure to ADSC-conditioned medium. These insights would shed light on mechanisms and likely lead to new lines of thought in alopecia treatment.

## Data Availability

Data supporting the findings of this study are available upon request from the corresponding author.

## Submission declaration

All authors have approved the submission of this manuscript.
